# Bitter Odorants and Odorous Bitters: Toxicity and Human TAS2R Targets

**DOI:** 10.1021/acs.jafc.3c00592

**Published:** 2023-06-01

**Authors:** Eitan Margulis, Tatjana Lang, Anne Tromelin, Evgenii Ziaikin, Maik Behrens, Masha Y. Niv

**Affiliations:** † Food Science and Nutrition, The Robert H Smith Faculty of Agriculture, Food and Environment, The Institute of Biochemistry, Food and Nutrition, The Hebrew University of Jerusalem, 76100 Rehovot, Israel; ‡ Leibniz Institute for Food Systems Biology at the Technical University of Munich, 85354 Freising, Germany; § Centre des Sciences du Goût et de l’Alimentation, CNRS, INRAE, Institut Agro, Université de Bourgogne Franche-Comté, F-21000 Dijon, France

**Keywords:** odor, olfaction, bitter, taste, toxic, machine learning, fishy, TAS2R14, GPCR, floral

## Abstract

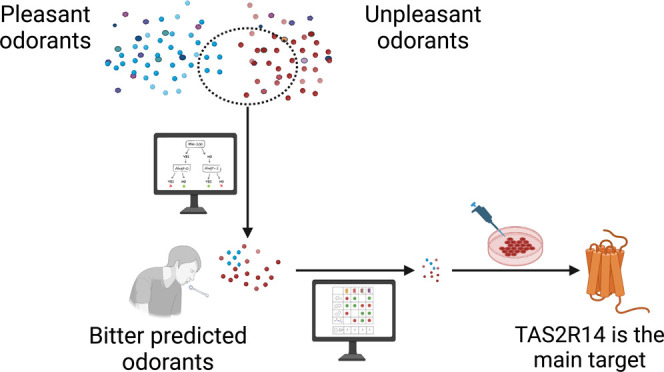

Flavor is perceived through the olfactory, taste, and trigeminal systems, mediated by designated GPCRs and channels. Signal integration occurs mainly in the brain, but some cross-reactivities occur at the receptor level. Here, we predict potential bitterness and taste receptors targets for thousands of odorants. BitterPredict and BitterIntense classifiers suggest that 3–9% of flavor and food odorants have bitter taste, but almost none are intensely bitter. About 14% of bitter molecules are expected to have an odor. Bitterness is more common for unpleasant smells such as fishy, amine, and ammoniacal, while non-bitter odorants often have pleasant smells. Experimental toxicity values suggest that fishy ammoniac smells are more toxic than pleasant smells, regardless of bitterness. TAS2R14 is predicted as the main bitter receptor for odorants, confirmed by *in vitro* profiling of 10 odorants. The activity of bitter odorants may have implications for physiology due to ectopic expression of taste and smell receptors.

## Introduction

The ability to respond to stimuli from the environment is one of the characteristics of living creatures.^1^ While physical signals such as light and sound are perceived through vision and hearing, chemical signals are perceived mainly by the senses of taste or smell.^2^ Chemosensation of molecules through taste or smell assists in the selection of nutritious foods and alarms against potentially spoiled or dangerous substances.^3,4^

The sense of taste is mediated by G-protein-coupled receptors (GPCRs) for the sweet, umami, and bitter taste modalities, and ion channels for salty and sour.^5−7^ It is generally known that at normal concentrations, compounds with sweet, salty, or umami taste are considered attractive while bitter and sour are aversive.^8^ Surprisingly, it appears that bitterness does not necessarily signal toxicity, as can be deduced, for example, from the lack of correlation between LD_50_ values and bitterness.^9^ Furthermore, there is an abundance of evidence that bitter compounds possess health-beneficial properties, such as antioxidative effects, anticancerous activities,^10^ and more.

More than a thousand molecules are known to elicit bitter taste, and ∼300 of the human bitter taste receptor (TAS2R) targets were established.^11^ In humans, there are 25 subtypes of TAS2Rs^12^ that are expressed not only in the oral cavity but in many extraoral tissues.^13^ Some receptors can be activated by a wide range of ligands while others are selective, having only 0–2 known ligands.^14^ Similarly, some bitter compounds can activate multiple receptors and some activate only a few.^14^ Bitter molecules are very diverse in their chemical structure and there are no simple rules to tell whether a compound is bitter or not, although bitterness is more common for hydrophobic rather than hydrophilic molecules.^15^ Thus, computational methods and machine learning models were developed to assist in the prediction of bitterness,^16^ intense bitterness,^17^ and the assignment to a specific TAS2R.^18^

The sense of smell is mediated by an even larger family of GPCRs, the olfactory receptors (ORs).^19^ ORs are encoded by more than 400 functional human genes.^20^ Unlike the very few basic modalities, it was suggested that humans can smell between 10,000 and 40 billion odors,^21^ emphasizing the complexity of this chemo-recognition system. Perception of this large magnitude of distinct smells is enabled by the odorants activating different combinations of ORs, which encode distinct odor identities;^22^ however, the connection between specific receptors to specific smells is frequently unclear. Interestingly, the physicochemical properties of odorous molecules correlate with their perceived odor and can be used to predict the pleasantness of an odorant.^23^ Moreover, one molecule can have different smells for different people, which can be due to genetic variations in the ORs,^24^ different rates of odor metabolism,^25^ the concentration of the odor, and in general the difficulty to describe an odor by words.^26^

Similarly to aversive taste, aversive odors can alert from consuming spoiled food^27^ or gas leakage. However, to the best of our knowledge, the connection between aversive odors and their toxicity has not been quantitatively studied.

Compounds that activate various types of chemosensory receptors or channels (olfactory, taste, and trigeminal) can contribute to the distinct flavor of foods and drinks. For example, vanillin, one of the most abundant flavoring agents in the world, is an odorant^28^ that acts via OR10G4^29^ (and maybe other ORs) and also activates several TAS2Rs^30^ and potentially TRP channels as well.^31^ α-Thujone is an odorant with cedar odor^32^ that activates human TAS2Rs 4 and 14,^33^d-camphor has minty camphoraceous odor,^32^ and activates TAS2Rs 4,10 and 14.^33^ Hence, a deeper understanding of odor–taste interactions at a molecular level is of interest for flavor design. In addition, olfactory receptors are not unique to the nose^34^ and bitter taste receptors are not unique to the tongue.^35^ Therefore, the cross-reactivity of odorants and bitterants may have physiological implications beyond flavor.

We hypothesize that some bitter molecules may have odors and may have distinct smell profiles, that there is a correlation between the unpleasantness of odorants (by taste, smell, or both) and toxicity values, and that there are particular TAS2R targets involved in identifying odorous bitterants.

To test our hypotheses, we analyze a dataset of odorants obtained from FlavorBase, a database of flavoring materials and food additives,^32^ and connect different odors to bitterness by using machine learning tools for bitterness prediction. We predict which odors have a bitter taste, and which are the TAS2Rs involved in their recognition. In addition, we predict which bitter molecules from the BitterDB^11^ may have odors, and elucidate the connection between aversion (by taste or smell) and toxicity by correlating LD_50_ values to bitterness and aversive smells.

## Materials and Methods

### Data Collection and Preparation

The odor descriptions of the odorants were obtained from the FlavorBase DB 9th edition,^32^ consisting of 3508 compounds with known odor notes. The chemical structures of the compounds were obtained from the work of Tromelin et al.^36^ The 3-dimensional structures were prepared using Maestro’s (Schrödinger Release 2021-1: MS Jaguar, Schrödinger, LLC, New York, NY, 2021) LigPrep and Epic (Schrödinger Release 2021-1: LigPrep, Epik, LLC, New York, NY, 2021). The compounds were prepared at pH 7 ± 0.5 and desalted when possible, keeping the bigger ion part of the compound and eliminating the smaller counter ion.

### Prediction of Bitterness and Bitterness Intensity

After the compounds were prepared in 3D, we calculated their chemical features using Canvas (Schrödinger Release 2019–2: Canvas, Schrödinger, LLC, New York, NY, 2019). We calculated three sets of features: physicochemical features, LigFilter features (moieties, atoms, and functional groups), and QikProp properties (ADME descriptors), in total 235 features were calculated for the prediction. For the QikProp descriptors, additional PM3 properties were calculated as well. Compounds that could not be neutralized were excluded from the sets due to the limitations of calculating QikProp descriptors.

The computed features were inputted into the BitterPredict^16^ algorithm for assigning the compounds into bitter and non-bitter and into BitterIntense^17^ algorithm to predict the bitterness intensity of the compounds. For BitterPredict, compounds that achieved a positive score were considered bitter-predicted, whereas a score of above 0.6 was predicted to be bitter in high confidence. Following previous work, a negative score suggested that the compound was non-bitter predicted and a score of −0.7 or below was considered not bitter in high confidence.^16^ Compounds that were outside the applicability domain based on their physicochemical properties were excluded from the prediction in BitterPredict.^16^ The list of compounds with BitterPredict scores and BitterIntense prediction probabilities can be found in the Supporting Information.

### Prediction of Odorous Bitterants

Prediction of odorous bitterants from BitterDB was performed using the rule of three.^21^ This rule states that compounds with a molecular mass between 30 and 300 Da and with fewer than three heteroatoms usually have an odor. These features were calculated for the compounds in BitterDB using the Python library RDKit (version 2022.09.3).

### Distribution of Bitter-Predicted and Non-Bitter-Predicted Compounds across Odor Categories

The distribution of bitter-predicted and non-bitter-predicted compounds was evaluated for pleasant and unpleasant odor categories for which bitterness/non-bitterness predictions were available. The categories with most of the bitter- and non-bitter-predicted compounds were chosen for evaluation. We tested the distribution of bitter- and non-bitter-predicted compounds in two ways: (1) by dividing the number of bitter (non-bitter)-predicted compounds in each odor category by the total number of bitter (non-bitter)-predicted compounds in our dataset. (2) by dividing the number of bitter- and non-bitter-predicted compounds in each odor category by the total number of odorants in that odor category. In addition, we performed statistical analysis to test the difference in proportions between bitter-predicted odorants across pleasant and unpleasant smells, using two-proportion Z-test, and significance was tested according to P<0.05.

### Common Scaffold Analysis

The common substructure of the bitter-predicted fishy-smelling compounds was extracted using the R-Group creator panel in Maestro (Schrödinger Release 2021-1). The R-groups were extracted by filtering the compounds sharing the same core according to SMARTs pattern. Specifically, the common scaffold was represented as [#6]–[#7]: carbon–nitrogen.

### Prediction of Target TAS2Rs

The prediction of target TAS2Rs was performed using the BitterMatch algorithm.^18^ Briefly, the algorithm predicts which of the 21 non-orphan human TAS2Rs are likely to be activated by the compounds. The algorithm makes the prediction by using the chemical features that were described above, and by including chemical similarities between the odorants and the bitter molecules in the training set of the algorithm, that were calculated using Canvas (Schrödinger Release 2019–2: Canvas, Schrödinger, LLC, New York, NY, 2019), based on linear fingerprints and MOLPRINT2D fingerprints. We considered the ligand-receptor match as positive (predicted activation) if the score was above 0.524 as described by Margulis et al.^18^

### Toxicity Analysis

The LD_50_ values of the odorants were collected from the NIH’s TOXNET^37^ database. We collected the values for oral administration in rats. In total, we obtained LD_50_ values for 498 compounds with fishy and pleasant odors (consist of floral, fruity, and sweet odorants). We computed the natural logarithm (*ln*) of the LD_50_ values since the distribution of the values was heavily skewed due to differences in the orders of magnitude of the values. The *ln* values scaled the data to fit into our statistical analysis.

Levene’s test was performed to verify the equality of variances between the groups, and no significant difference was observed in the analysis. The difference between the ln(LD_50_) values was evaluated using a two-tailed *t*-test and ANOVA, *P* < 0.05.

Classification of compounds to the toxicity categories was done according to Nissim et al.^9^ which is based on the United Nations Globally Harmonized System (GHS) classification and labeling of chemicals, revision 6.^38^

### Chemicals and Materials

All test compounds listed in Table S1 were dissolved as stock solutions in dimethyl sulfoxide (DMSO) to 100 mM and stored at −20 °C until use. For the assays, stock solutions were diluted in C1 buffer (130 mM NaCl, 5 mM KCl, 2 mM CaCl_2_, 10 mM glucose, 10 mM HEPES; pH 7.4). The final DMSO concentration in the experiments did not exceed 1%. Depending on the limited solubility of the compounds in the C1 buffer or artifacts during the measurement, the final experimental concentrations were between 0.1 and 0.3 mM (Table S1).

### Functional Calcium Mobilization Assay

Screening of test compounds and determination of the dose–response relationships of TAS2R agonists were performed analogously to previous publications.^39^ Briefly, HEK 293T-Gα16gust44 cells were grown on poly-d-lysine-coated 96-well plates under regular conditions (DMEM, 10% FCS, 1% penicillin/streptomycin, 1% glutamine; 37 °C, 5% CO2, 95% humidity) and transiently transfected with cDNA constructs coding for the 25 TAS2Rs, respectively, using Lipofectamine 2000 (Thermo Fisher Scientific). An empty vector (mock) was used as a negative control. After 24 hours of incubation, the cells were loaded with the calcium-sensitive dye Fluo4-AM (Thermo Fisher Scientific) and probenecid (2.5 mM, Sigma-Aldrich) for 1 h. After the second wash with C1 buffer to remove excess Fluo4-AM, the cells were placed in a fluorometric imaging plate reader (FLIPR^Tetra^, Molecular Devices). Test compounds were automatically administered to the cells. Aristolochic acid was used as a positive control for TAS2R14^40^ and strychnine for TAS2R10^41^ and TAS2R46,^42^ respectively. Before and after application, the changes in fluorescence (at 510 nm excitation and at 488 nm emission) were recorded. Finally, cell viability was tested by the application of somatostatin 14 (100 nM, Bachem). Determination of dose–response relationships was performed in three independent experiments, each in duplicate wells. For calculation of the compound-specific fluorescence changes (Δ*F*/*F*), mock fluorescence was subtracted and normalized based on background fluorescence. The plots were generated in SigmaPlot 14.0.

### Data Analysis and Graphics

All of the data were analyzed using Python 3.8.16, including the packages: Pandas (1.3.5), NumPy (1.21.6), and SciPy (1.7.3). The figures were obtained by using Matplotlib (3.2.2), seaborn (0.11.2), and BioRender (www.Biorender.com).

## Results

### Bitterness Prediction of Odorants and Potentially Odorous Bitterants

**Figure 1:**
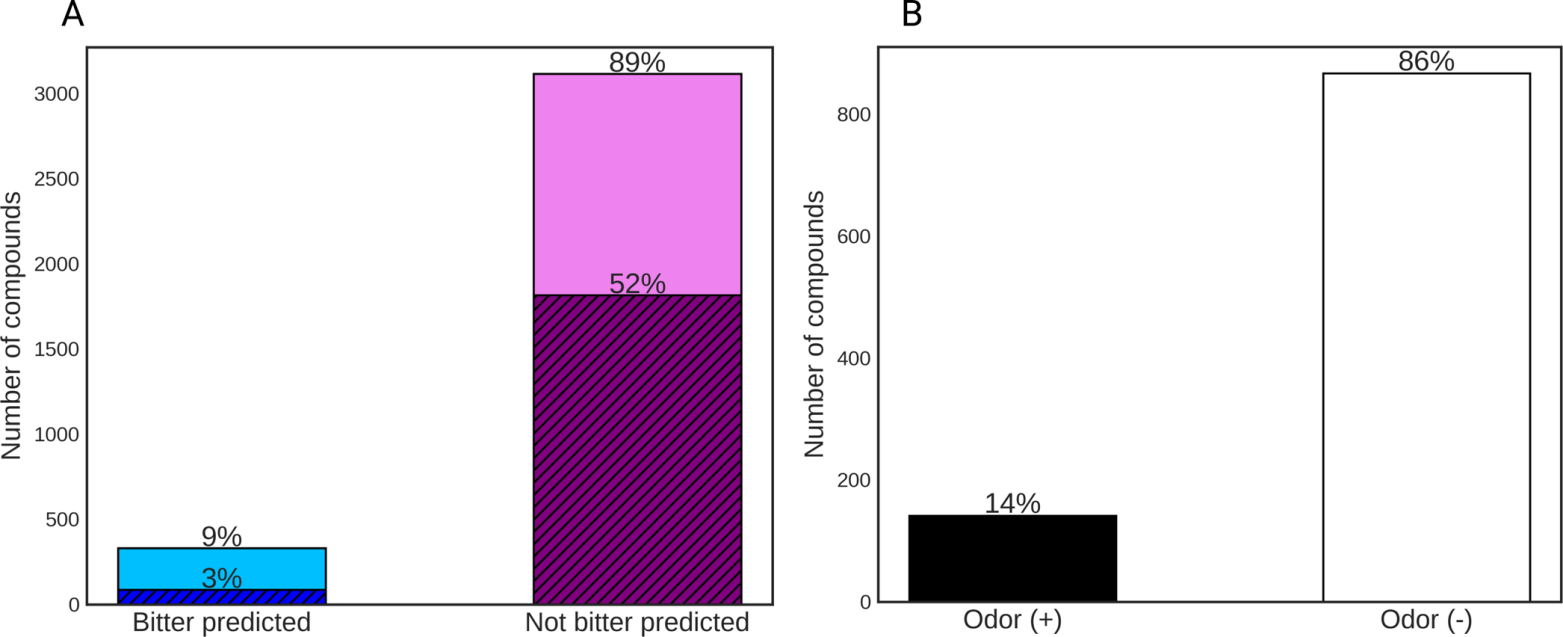
Computational prediction of bitterness and odor. (A) Bitterness prediction of odorants by BitterPredict model. The bitter-predicted compounds are represented in the blue bar, whereas the hatched dark blue color represents the high-confidence predictions. In purple are the non-bitter-predicted compounds, whereas the hatched dark purple color represents the high-confidence predictions. The percentages of each group appear on top of each bar. 1.8% of compounds could not be assigned because they were out of the applicability domain of the predictive model. (B) Prediction of odorous compounds among bitter molecules in BitterDB. Odor(+) represents the compounds that comply with the rule of three and potentially have an odor. Odor(−) represents the compounds that were not predicted to have an odor because they do not follow the rule of three.

Bitterness prediction was performed on a dataset of 3508 odorants from FlavorBase DB,^32^ using BitterPredict^16^ and BitterIntense^17^ algorithms (see the Methods section). Briefly, BitterPredict is a machine learning classifier that can assign compounds to “bitter” or “not bitter” according to their chemical structure. BitterIntense was used to predict intensely bitter compounds and assign them as “very bitter” or “not very bitter”. In order to make predictions with both algorithms, we calculated the 3D structures of the molecules as well as their chemical properties, including physicochemical properties, functional groups and atom types, and pharmacological properties (see the Methods section). BitterPredict was able to make a prediction for 3445 compounds in the applicability domain of the classifier, the predictions (Tables S2 and S5) suggested that the majority of the odorants do not have a bitter taste (89%), where 52% are predicted to be not bitter in high confidence (Figure 1A). Only 9% are predicted to be bitter, and 3% are predicted to be bitter in high confidence (Figure 1A). BitterIntense predictions suggested that only 10 compounds (less than 0.3%) are predicted to be intensely bitter (Tables S3 and S6).

In addition, we predicted how many out of the 1008 unique known bitter compounds from BitterDB^11^ have the potential to be odorous. Predicting odorous bitterants using the recent transport features model^21^ resulted in an unrealistically high number of predicted compounds, suggesting a potential incompatibility of the chemical space with the model’s applicability domain. We therefore applied the rule of three,^21^ which states that molecules with molecular mass between 30 and 300 Da and with fewer than three heteroatoms generally have an odor. After applying the rule, we have also eliminated obvious false positives such as salts. This resulted in 138 (14%) bitter compounds that were predicted as odorous (Figure 1B, Table S4), and the remaining 870 (86%) as non-odorous (Figure 1B).

These predictions suggest that almost no odorants are intensely bitter, but 3–9% are expected to have a bitter taste, and 14% of bitter molecules may have an odor.

### Distribution of Bitter-Predicted and Non-Bitter-Predicted Odorants across Smell Categories

**Figure 2:**
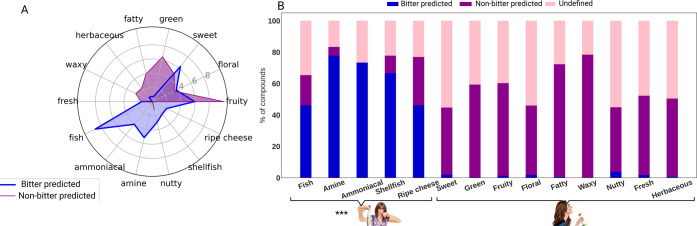
(A) Distribution of bitter- and non-bitter-predicted odorants across different odor categories. For each smell category, odorants were predicted by BitterPredict to be bitter or not. Results are presented in percentages that were normalized to the sizes of bitter- and non-bitter-predicted groups, where each group is 100%. (B) Percentage of bitter and non-bitter compounds in each odor category. The odorants in each category were divided into bitter-predicted (blue), non-bitter-predicted (purple), and undefined (pink). The undefined groups are compounds that were outside the applicability domain of BitterPredict. The percentages were calculated by dividing the number of bitter or non-bitter by the total number of compounds in each odor category. The statistically significant difference in the proportions of bitter-predicted compounds between pleasant and unpleasant odors was observed using two-proportion *Z*-test (*z* = 36.5412, *P* < 0.00001).

Each odorant in the dataset contained several smell descriptions (out of the 251 smell descriptions that were used in FlavourBase DB^32^). We compared the smell categories that are abundant for bitter-predicted odorants and non-bitter-predicted odorants. Different distributions of bitter- and non-bitter-predicted odorants across smells may imply that bitterness can be associated with specific smells. We analyzed the distributions in two manners: (1) by dividing the number of bitter (or of non-bitter)-predicted compounds in each odor category by the total number of bitter (or non-bitter)-predicted compounds in the dataset (Figure 2A); (2) by dividing the number of bitter- and non-bitter-predicted compounds by the total number of odorants in this odor category (Figure 2B). Our results (Figure 2A) suggest that the most common smell for bitter-predicted odorants is the fishy smell (9% of bitter compounds), followed by sweet (6.3%), fruity (6%), amine (5.2%), and ammoniacal (4%) smells (Figure 2A). However, the most common smells for non-bitter-predicted compounds are fruity smell (10%), green (6.5%), sweet (5%), fatty (4%), and floral (3.4%). When comparing the distributions across smells, the results suggest that bitter-predicted compounds tend to have more unpleasant odors such as fish and amine in comparison to non-bitter predicted odorants (Figure 2A). To further test our hypothesis that bitter compounds are associated with bad smells, we analyzed the distribution of bitter- and non-bitter-predicted compounds within several pleasant and unpleasant smell categories that had most of the bitter- and non-bitter-predicted compounds (Figure 2B). The results suggest that the proportion of the bitter-predicted compounds among unpleasant odors (61 out of 107 compounds) is significantly higher than bitter-predicted compounds among pleasant odors (61 out of 4779). Bitter-predicted compounds are dominant among unpleasant odors such as fishy (46%), amine (78%), ammoniacal (73%), shellfish (67%), and ripe cheese (46%), while non-bitter compounds are dominant in pleasant odors such as sweet (42%), green (59%), fruity (59%), and floral (59%). The sweet and fruity were also in the top categories for bitter-predicted compounds (Figure 2A); however, when considering bitter/non-bitter proportion within these odor categories, bitter-predicted compounds were much less abundant, with 2 and 1%, respectively (Figure 2B). This result implies that unpleasant smells are more often bitter than pleasant smells. Nevertheless, we note that the 10 intensely bitter-predicted compounds did not have fishy-like odors, but rather oily, fruity, and floral notes.

**Figure 3:**
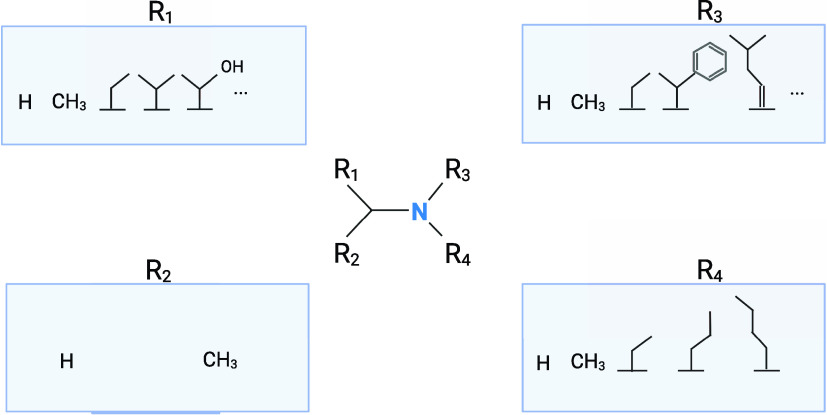
Common scaffold for fishy, amine, and ammoniacal odorants. The amine scaffold is shared between 34 compounds out of 57 fishy, amine, and ammoniacal-smelling compounds. The detailed R-groups appear in boxes, where for R1 and R3 there are additional optional groups that are not represented.

Analysis of the chemical structures revealed that amines (in particular tertiary amines) and positively charged nitrogens are common in fishy, amine, and ammoniacal odorants. Out of 57 compounds with fishy, amine, and ammoniacal smells, 34 had a common scaffold of an amine group (Figure 3), where 22 were tertiary amines and the rest of the compounds contained a different type of amines (including ammonium ions). Structural search in BitterDB revealed that 255 bitter compounds have tertiary amines, 317 have secondary amines and 94 have primary amines, suggesting that this is a common feature for bitter and fishy odorants.

### Toxicity Analysis of Odorants with Unpleasant Smells and Bitter Taste

**Figure 4:**
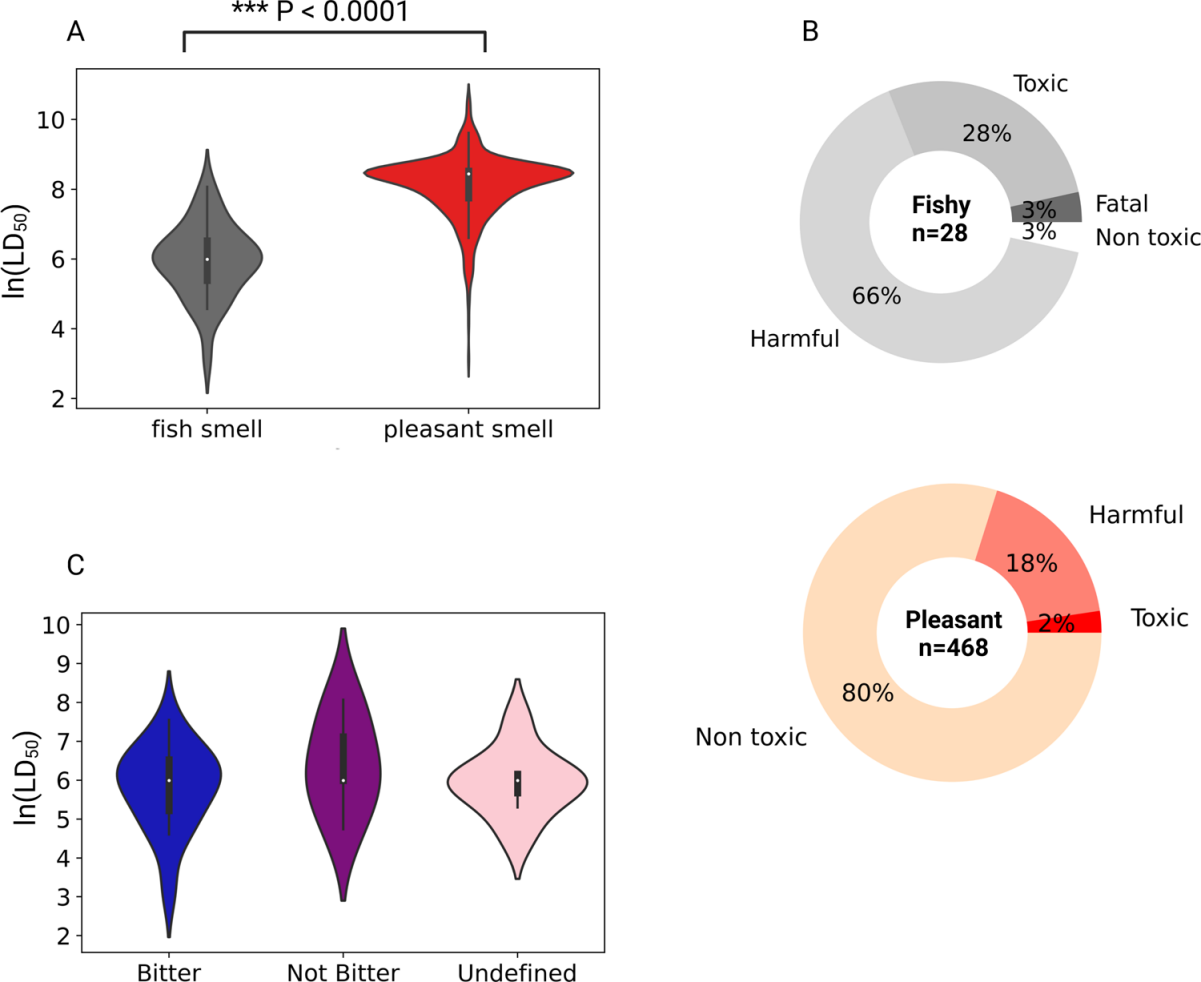
Toxicity analysis of odorants. (A) Comparison between ln(LD_50_) values of fishy-smelling odorants (gray) and pleasant odorants consists of floral, fruity, and sweet odorants (red). (B) Classification of fishy and pleasant odorants to the toxicity categories: “Fatal” (LD_50_ < 50 mg/kg bw), “Toxic” (LD_50_ 50–300 mg/kg bw), “Harmful” (LD_50_ 300–2000 mg/kg bw), “Non toxic” (LD_50_ >2000 mg/kg bw). (C) Comparison between ln(LD_50_) values of fishy-smelling odorants with a bitter-predicted taste (blue), non-bitter-predicted taste (purple), and undefined taste (pink). Levene’s test was performed to verify the equality of variances between groups, and no significant difference was observed in the analysis (*P* > 0.05). The difference between the ln(LD_50_) values was evaluated using a two-tailed *t*-test and ANOVA, *P* < 0.05.

The conclusion that bitterness might be associated with unpleasant odors raises a question regarding toxicity. Are aversive smells usually elicited by toxic compounds? And if a compound is aversive by both smell and taste, does it necessarily mean that it is also toxic, as a protective mechanism from consuming these substances? In order to answer these questions, we collected the median lethal dose (LD_50_) values for the compounds from NIH’s TOXNET^37^ database (mg/kg, oral administration in rats, see the Methods section). First, we compared the available ln(LD_50_) values of 28 compounds with fishy odors (the most enriched group with bitter compounds) to those of 468 compounds with pleasant odors (floral, fruity, and sweet). The median LD_50_ value for fishy odorants is 400 mg/kg bw (ln(LD_50_) = 6), and the median for pleasant odorants is 4650 mg/kg bw (ln = 8.44). The results suggest that the fishy-smelling odorants have significantly lower ln(LD_50_) values than the ln(LD_50_) values of pleasant odorants meaning that fishy-smelling odorants tend to be more toxic than pleasantly smelling odorants (Figure 4A). The same result was achieved when comparing between the fishy ln(LD_50_) values and each of the pleasant odor categories separately (Figure S2). In addition, we classified the odorants to toxicity categories as was previously done by Nissim et al.^9^ (Figure 4B). The classification suggests that most of the fishy odorants are found in the “Harmful” (66%) and “Toxic” (28%) categories, while the pleasant odorants are mostly nontoxic (80%). We further investigated whether bitter-predicted fishy odorants are more toxic than non-bitter-predicted fishy odorants, finding no significant differences between the toxicity values (Figure 4C). No significant difference was found also for ln(LD_50_) values of bitter- and non-bitter-predicted odorants with pleasant smells (Figure S3).

Together, our results suggest that fishy-smelling compounds tend to be more toxic than pleasant odorants, and bitterness does not further contribute to this difference.

### Computational Assignment of Bitter Odorants to TAS2Rs

**Figure 5:**
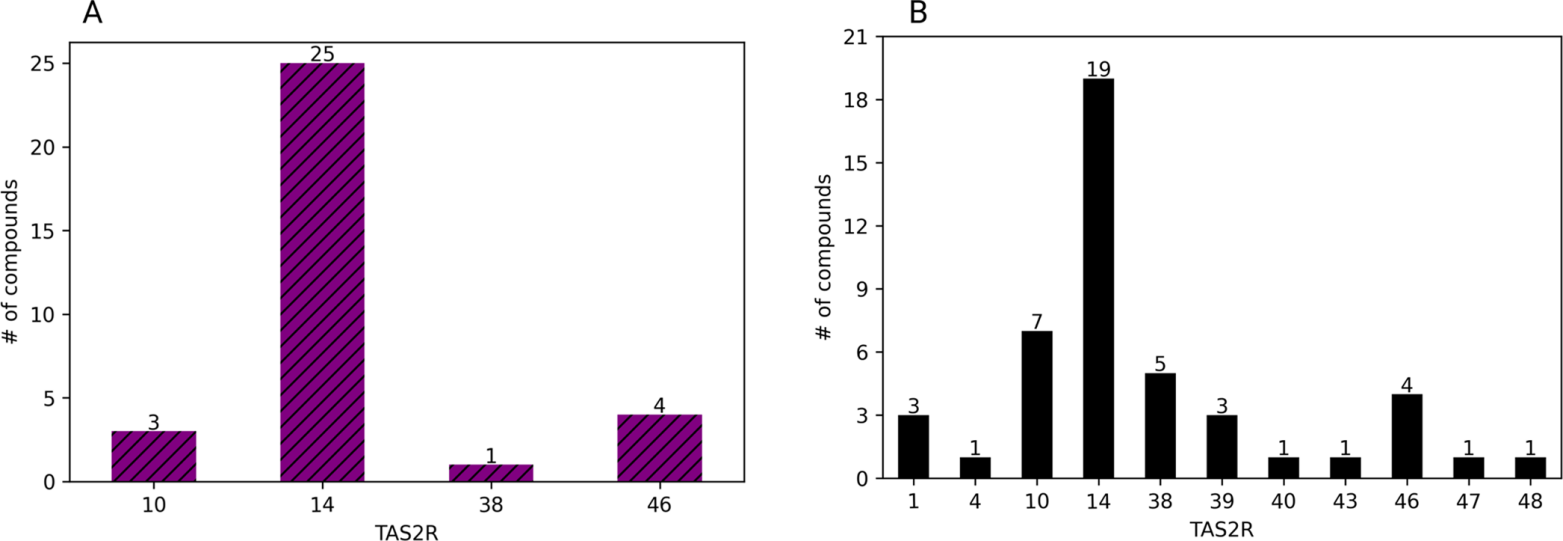
Matching odorants to bitter taste receptors. (A) Number of compounds from FlavorBase DB that were predicted as bitter by BitterPredict and matched to individual receptors with BitterMatch. (B) Experimentally determined TAS2R targets for potentially odorous bitter molecules.

Since we predicted that 3–9% of odorants are bitter, we set out to identify their potential TAS2R targets. We applied the BitterMatch algorithm^18^ to the FlavorBase DB^32^ (see the Methods section). The algorithm predicts which human TAS2Rs (out of 21 non-orphan) may be activated by the compounds. Each ligand–receptor pair that is predicted to associate (meaning that the ligand is activating the receptor) is considered a positive prediction. Briefly, in order to make the prediction, we used the chemical features that were calculated for the bitterness prediction, and in addition, we calculated similarities between the odorants and the training set of the BitterMatch to create the similarity-based features.^18^ After combining the predictions of BitterPredict with BitterMatch, 33 compounds were predicted both as bitter in high confidence and were matched to at least one TAS2R (Figure 5A). Three compounds were predicted to activate TAS2R10, 25 compounds were predicted to activate TAS2R14, one compound was predicted to activate TAS2R38, and four were predicted to activate TAS2R46. In addition, we also collected the available experimentally determined associations of 28 potentially odorous bitter compounds to their target TAS2Rs from the BitterDB^11^ (Figure 5B). The results suggest that TAS2R14 is the main bitter target for all of these compounds, with TAS2Rs 46, 38, and 10 also frequently predicted. BitterDB compounds that are predicted to have odor had also additional TAS2R targets, a difference that could be due to small sample sizes, potential errors in BitterMatch, as well as errors in predicting whether a molecule is an odorant.

### *In Vitro* Testing of Computationally Predicted Associations of Odorants with Bitter Taste Receptors

Following BitterPredict and BitterMatch analysis of FlavorBase DB, we selected 9 odorants out of the 33 that were predicted to be bitter and assigned to at least one TAS2R with high confidence, to test their ability to activate the 25 human TAS2Rs in functional cell assays (see the Methods section). We chose only substances that had no offensive smell in order to avoid contact of co-workers not involved in the study with polluted air since full containment of equipment was not possible. A pleasantly smelling molecule, 2,6,10,14-tetramethylpentadecane, was chosen as a control, as it was predicted by BitterPredict to be non-bitter. Thus, 10 compounds in total were tested *in vitro*, amounting to 30% of the predictions.

**Figure 6:**
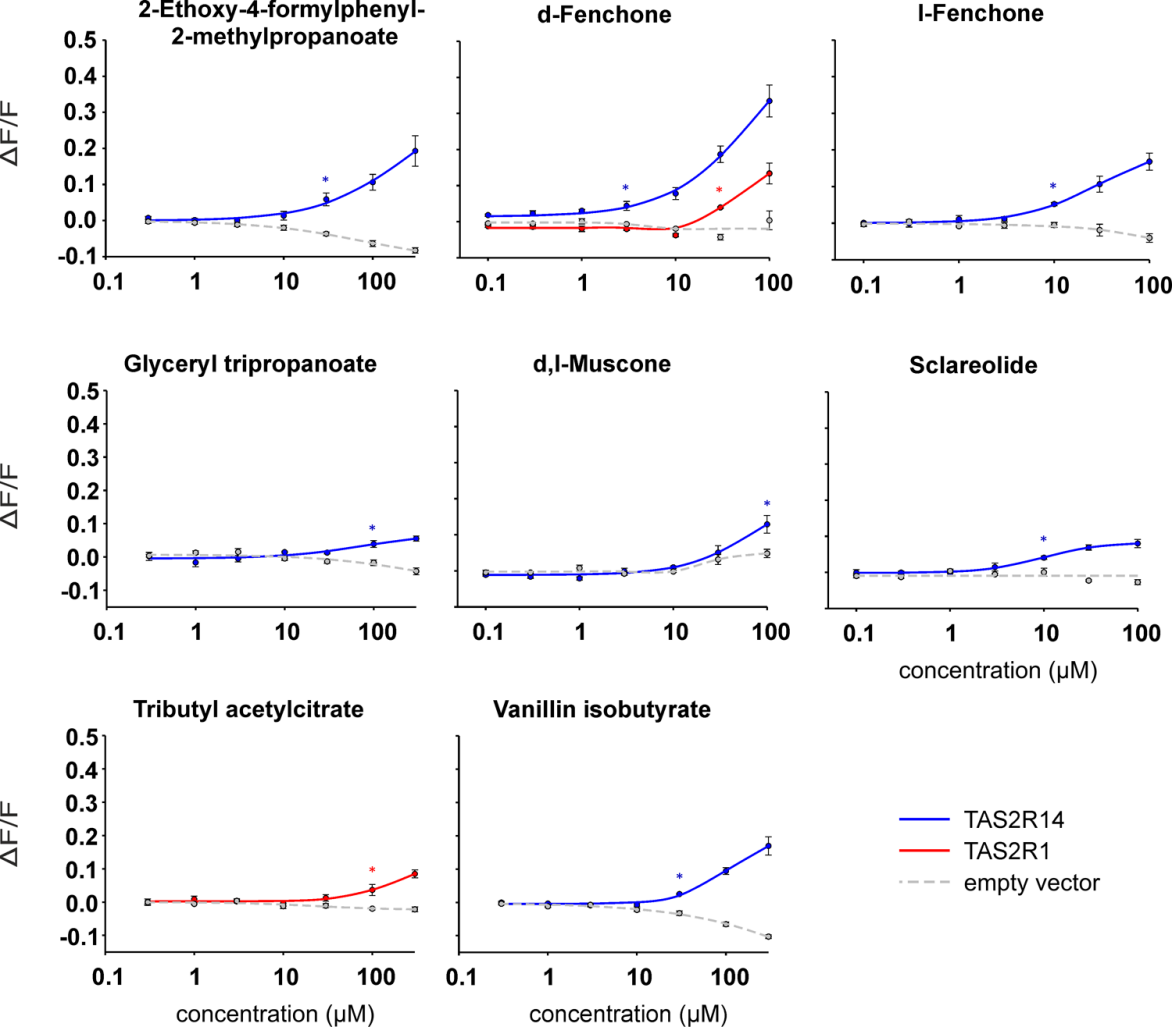
Dose–response relationships of TAS2Rs activating odorants. TAS2R14 (blue), TAS2R1 (red), or mock transfected cells (gray) were challenged with increasing concentrations of odorants. Automated odorant application and fluorescence measurements were done using a fluorometric imaging plate reader (FLIPRtetra). The relative changes in fluorescence (DF/F) were plotted on the *y*-axes, and the concentrations of the compounds in μM were plotted on the logarithmically scaled *x*-axes. Asterisks indicate the lowest concentrations leading to statistically significant higher signals in receptor transfected cells compared to identically treated empty vector (mock) transfected cells (defined as threshold concentrations). The significance was tested using Student’s *t*-test, *P* < 0.05.

Our experimental results showed that 8 out of the 10 selected compounds activated TAS2Rs (Figures 6 and S1). In accordance with our predictions (Table 1), the functional assays confirm that TAS2R14 is the main receptor for detecting the tested odorants (Table 1), where 7 out of the 10 tested compounds were TAS2R14 agonists. In addition, 2 compounds (d-fenchone and tributyl acetylcitrate) were not predicted to do so but experimentally activated also TAS2R1. The control compound 2,6,10,14-tetramethylpentadecane was predicted as non-bitter by BitterPredict and indeed did not activate any of the 25 human TAS2R receptors at the tested concentrations. Overall, all of the tested concentrations of the compounds were comparable with the lowest reported flavor detection concentrations (Table S7).

**Table 1:**
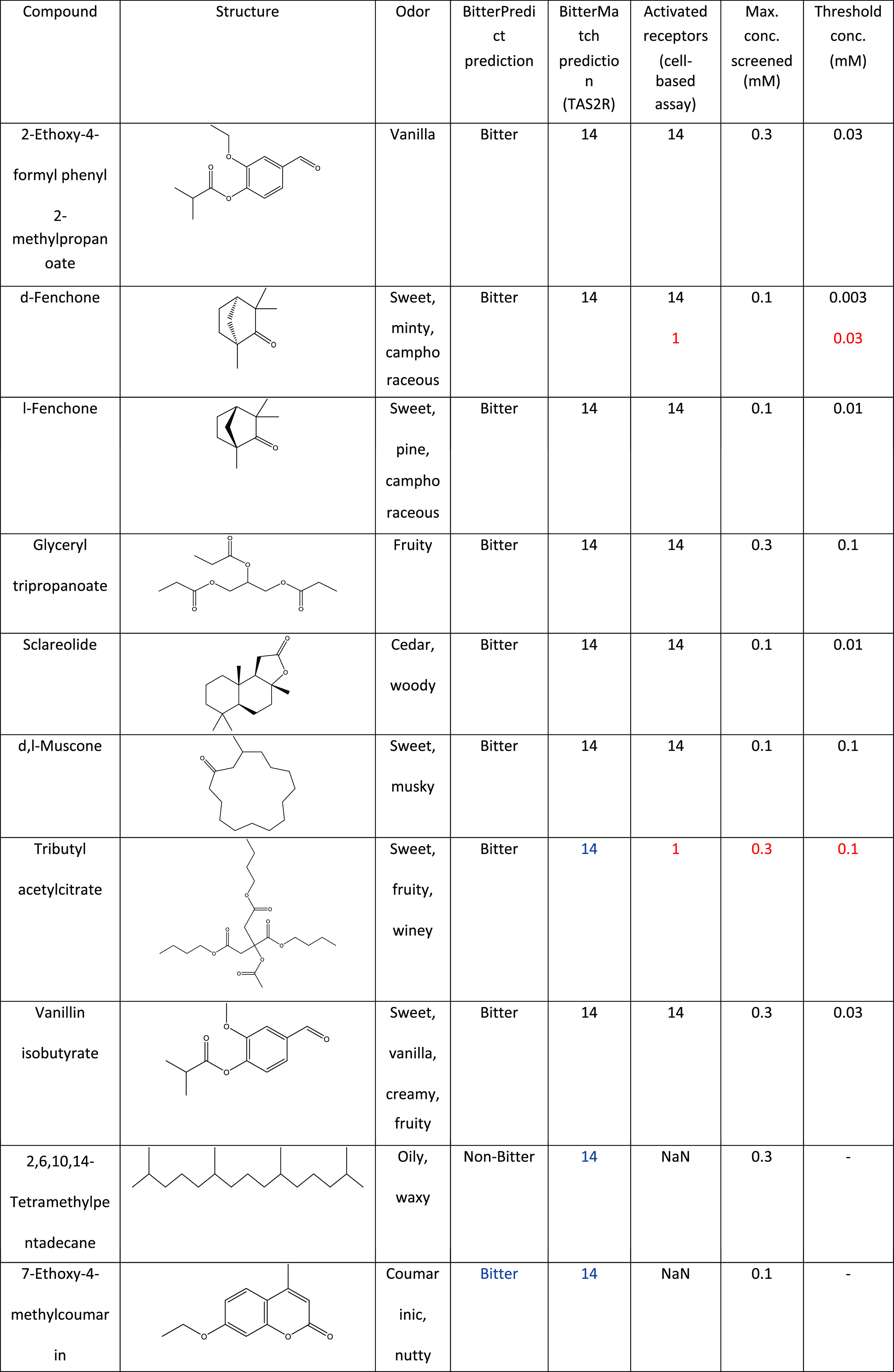
Summary of Experimental Results for Computational Predictions of TAS2R Activation by Odorants^a^

a False-positive predictions are in dark blue, false negatives are in red.

When comparing the maximal signal amplitudes obtained for TAS2R14 with 10 μM aristolochic acid (a known ligand of TAS2R14,^40^ which served as a positive control), we observed that d-fenchone stimulation of TAS2R14 transfected cells reached almost the same signal amplitude, and may thus be considered as a full agonist (Figure S1). In contrast to that, the other agonistic volatiles activating TAS2R14 may represent partial agonists (Figure S1).

The performance of the machine learning algorithms is summarized in Table 1: BitterPredict identified correctly 9 out of 10 compounds (8 true positives, 1 true negative, and 1 false positive) achieving 90% accuracy with 89% precision and 100% recall. 7-Ethoxy-4-methylcoumarin was predicted to be bitter; however, perhaps due to the limited solubility of maximally 0.1 mM, we did not observe activation of any of the receptors in the cell assay. Overall, BitterMatch predicted the association of the 10 odorants to 21 non-orphan TAS2Rs (210 predicted pairs): 7 ligands were correctly assigned to TAS2R14, 198 pairs were correctly identified as negatives (true negatives), 3 ligands were assigned incorrectly as TAS2R14 agonists (false positives), and 2 pairs were missed as TAS2R1 agonists (false negatives). In total, BitterMatch achieved an accuracy of 98% (balanced accuracy is 88%) with a precision of 70% and recall of 78%, and TAS2R14 experimentally supported as the most important bitter receptor target of odorant molecules. TAS2R1 appeared twice as false negative, and therefore may represent a potential target as well. Our results in Figure 5B indeed indicated that TAS2R1 is a potential target for some odorants; however, BitterMatch did not catch these associations.

## Discussion

In this work, we analyzed the connection between bitterness and smell. Bitter compounds are known to have diverse chemical structures with a molecular mass that ranges between 27 and 1524 g/mol, whereas large bitter compounds with many heavy atoms are known to be intensely bitter.^17^ The binding site of TAS2Rs is large relative to other GPCRs,^14,40^ accommodating diverse sets of ligands, among them large organic compounds and peptides. Odorants are usually known to be small volatile compounds with a molecular mass below 300–400 g/mol.^43^ We, therefore, did not expect a large number of odorants to activate bitter taste receptors. We used a machine learning algorithm, “BitterPredict”^16^ to identify how many odorants from FlavorBase, a database of flavoring materials and food additives, are predicted to have a bitter taste. Our results suggest that out of 3508 odorants, only about 2.5% are predicted to be bitter in high confidence, in agreement with our expectation.

Inversely, we applied the rule of three^21^ on bitterants from BitterDB to predict how many bitter compounds are expected to have an odorous characteristic. This set of rules describes two physicochemical properties that determine roughly whether a compound should have a smell. The analysis suggests that ∼14% of the known bitter compounds may also have a smell. This result implies that our previous conclusion applies both ways: most of the odorants do not have a bitter taste and most of the bitter compounds do not have a smell.

When comparing the odors of bitter- and non-bitter-predicted odorants, we discovered that bitter-predicted odorants are distributed among pleasant and unpleasant smells, mainly fishy amine sweet, and fruity. However, the non-bitter-predicted odorants mainly have pleasant odors. In addition, by looking at each of the odor categories and testing the distribution of bitter- and non-bitter-predicted odorants, we confirmed that unpleasant smells, such as fishy, amine, ammoniacal, shellfish, and ripe cheese, are enriched with bitter-predicted compounds, while pleasant smells such as sweet, green, fruity, and floral are enriched with non-bitter-predicted compounds. The fact that the BitterPredict identified overlaps between unpleasant smells with bitter molecules suggests a chemical similarity between these two groups. Indeed, while amine groups (with tertiary amine groups and positively charged nitrogens in particular) are common among these odorants, they are also found in many bitter compounds in BitterDB.^11^ These results hence suggest that unpleasant smells can be accompanied by unpleasant tastes, while pleasant smells are usually not aversive by taste. Several compounds with amine groups and fishy or amine smells are commonly found in spoiled foods.^44^ For example, diethylamine^45^ and pyrrolidine^46^ are markers for fish and seafood spoilage and are also predicted to be bitter by BitterPredict.^16^ Also, piperidine, which is known to be bitter^11^ and has a urine-like ammoniacal odor, is a metabolite that is produced in spoiled wines by bacteria.^47^ Interestingly, the ammonium ion was shown to inhibit T cell growth and impact immunotherapy,^48^ and the potential effects of ammonium ion on ORs and TAS2Rs (which are often expressed in tumors^49^) require further study.

To test relevance for toxicity detection, we analyzed the toxicity values (LD_50_, oral administration in rats) of the fishy smells category (containing most of the bitter-predicted compounds) and of pleasantly smelling odorants. Our results indicate that smell is a better marker for toxicity than bitterness since fishy compounds had significantly lower LD_50_ values than pleasant odorants, which indicates that they are more toxic. Comparing the bitter- and non-bitter-predicted fishy odorants, we found that bitterness does not further contribute to the toxicity of the compound. This means that if a compound smells fishy, it is more likely to be toxic; however, if it is also bitter, it is not necessarily more toxic than the non-bitter fishy odorant. Bitterness, despite common belief, was shown to be a poor marker of toxicity,^9^ and our results confirm this also in the context of odorants.

To the best of our knowledge, there is no current analysis of smell categories and LD_50_ values, and our work is the first to suggest such a correlation. It is important to note that while fishy compounds have significantly lower LD_50_ values than pleasant odorants, they are in general not highly poisonous. Rather, our analysis suggests that most of the fishy compounds are harmful or toxic, but not fatal. For comparison, the median LD_50_ value for fishy compounds is 400 mg/kg bw, the most toxic fishy-smelling compound has an LD_50_ value of 25 mg/kg bw, while the rat poison strychnine has an LD_50_ of 2.35 mg/kg bw, and the highly consumed coffee ingredient caffeine has LD_50_ of 192 mg/kg bw.

We next predicted and tested which TAS2Rs are prone to recognize bitter-predicted odorants. Our results suggest that the dedicated receptor for odorants is TAS2R14, a broadly tuned receptor that has hundreds of known ligands, including drugs and natural compounds.^11^ Due to its promiscuity, we expected that some compounds will activate TAS2R14. However, we were surprised by the specificity of these ligands toward TAS2R14, since 6 out of 8 bitter odorants activated only TAS2R14 and no other TAS2R. In addition, the results imply that TAS2R1 is also a target of some odorants, which was unexpected since TAS2R1 is known to be activated vastly by peptides and some natural products,^11^ which are much bigger than the small volatile odorants. This also might be the reason why BitterMatch algorithm, which overall performed very well, has missed these associations. Therefore, this type of data will be used to improve the BitterMatch next version.

Our *in vitro* results suggest that the tested molecules were relatively weak agonists, with threshold concentrations ranging from 3 μM (d-fenchone, TAS2R14) to 100 μM (glyceryl tripropanoate; d,l-muscone; tributyl acetylcitrate; all TAS2R14) and maximal signal amplitudes between 0.055 (glyceryl tripropanoate, TAS2R14) and 0.334 (d-fenchone, TAS2R14), in accordance with the small sizes of the molecules. In fact, we observed d-fenchone stimulation of TAS2R14 transfected cells almost reached the same signal amplitude as the positive control, and may thus be considered as full agonist.

d-Fenchone was described previously as “somewhat bitter” in Fenaroli’s Handbook of Flavor Ingredients;^11^ however, the TAS2R targets were unknown. BittterMatch predicted TAS2R14 for both isomers, and that was indeed confirmed *in vitro*. However, TAS2R1 was not predicted as a target and was shown here *in vitro* to be activated by d-fenchone, but not by l-fenchone. This is an example of how a change at one chiral center can change the biological activity, and the potential of such data to further improve computational models.

The dual effects of bitter odorants on ORs and TAS2Rs might have implications for flavor design for food and may also have physiological implications since TAS2Rs and ORs are known to be expressed in extraoral^35^ and extranasal^34^ tissues. It was previously shown that activation of TAS2Rs in the respiratory system might help in the case of asthma by promoting relaxation of the airway smooth muscles and also elicits an immune response in the presence of quorum sensing molecules secreted by bacteria.^50^ Thus, the discovery of specific volatile bitterants with high affinities might be relevant for the development of new inhaled drugs for treating symptoms of asthma or assisting to fight bacterial infection in the respiratory systems.

There are several limitations to our study. First, we keep in mind that most of our results are based on predictions made by models, and so while we can conclude the general trend, we would also expect some mistakes (both false positives and false negatives). For example, since the major limitation of the rule of three is that it is very general, we expect more false-positive predictions and so the number of odorous-predicted bitterants might be overestimated. Second, the toxicity analysis was performed only on compounds with available LD_50_ values (26% of fishy, floral, sweet, and fruity compounds) since experimental LD_50_ values are lacking for the rest, and the reported trend might change with additional data. Furthermore, LD_50_ refers to lethal doses and is measured in rats, but toxicity could be measured in other ways that do not result in death (NOAEL, hepatotoxicity, cardiotoxicity, and more) and may differ between rats and humans. Correlating these types of toxicities may provide additional insights regarding the toxicity of odorants and the effect of bitterness. Third, only 10 odorants were tested in our study (30% of the predictions), and with additional testing, more TAS2R targets of odorants might emerge. In addition, because of experimental limitations and safety issues, fishy-smelling compounds were not experimentally tested with TAS2Rs and are of interest for future work.

Our work highlights a ligand–receptor level of cross-reactivity between bitter taste and smell, contributing molecular-level insights into the multilayered complexity of flavor. We found connections between aversive bitter taste and aversive fishy smell, and a correlation between smell quality and toxicity levels as deduced from LD_50_ values. This paves the way for additional receptor-based research on off-flavors and future applications in food and pharma applications.

## Supporting Information Available

Lists of predicted compounds (bitter-predicted, odorous-predicted, and intensely bitter-predicted), as well as additional figures and experimental data (PDF)
Full tables with the predictions scores and probabilities for all of the odorants data as well as the flavor threshold of the tested compound (XLSX)
