# Supplementary material for: Bitter Odorants and Odorous Bitters: Toxicity and Human TAS2R Targets
Source: J Agric Food Chem. 2023 Jun 1;71(23):9051–61. doi: 10.1021/acs.jafc.3c00592 (PMC10273317; doi:10.1021/acs.jafc.3c00592)
Supplement: Supplementary file 1 — jf3c00592_si_001.pdf [file jf3c00592_si_001.pdf]

**Supporting Information**

# Bitter odorants and odorous bitters: toxicity and human TAS2R targets

Eitan Margulis[1], Tatjana Lang[2], Anne Tromelin[3], Evgenii Ziaikin[1], Maik Behrens[2], Masha Y. Niv[1*]

1. The Institute of Biochemistry, Food Science and Nutrition, The Robert H Smith Faculty of Agriculture, Food and Environment, The Hebrew University of Jerusalem, 76100, Rehovot, Israel.

2. Leibniz Institute for Food Systems Biology at the Technical University of Munich, 85354, Freising,

Germany.

3. Centre des Sciences du Goût et de l'Alimentation, CNRS, INRAE, Institut Agro, Université de Bourgogne Franche-Comté, F-21000 Dijon, France.

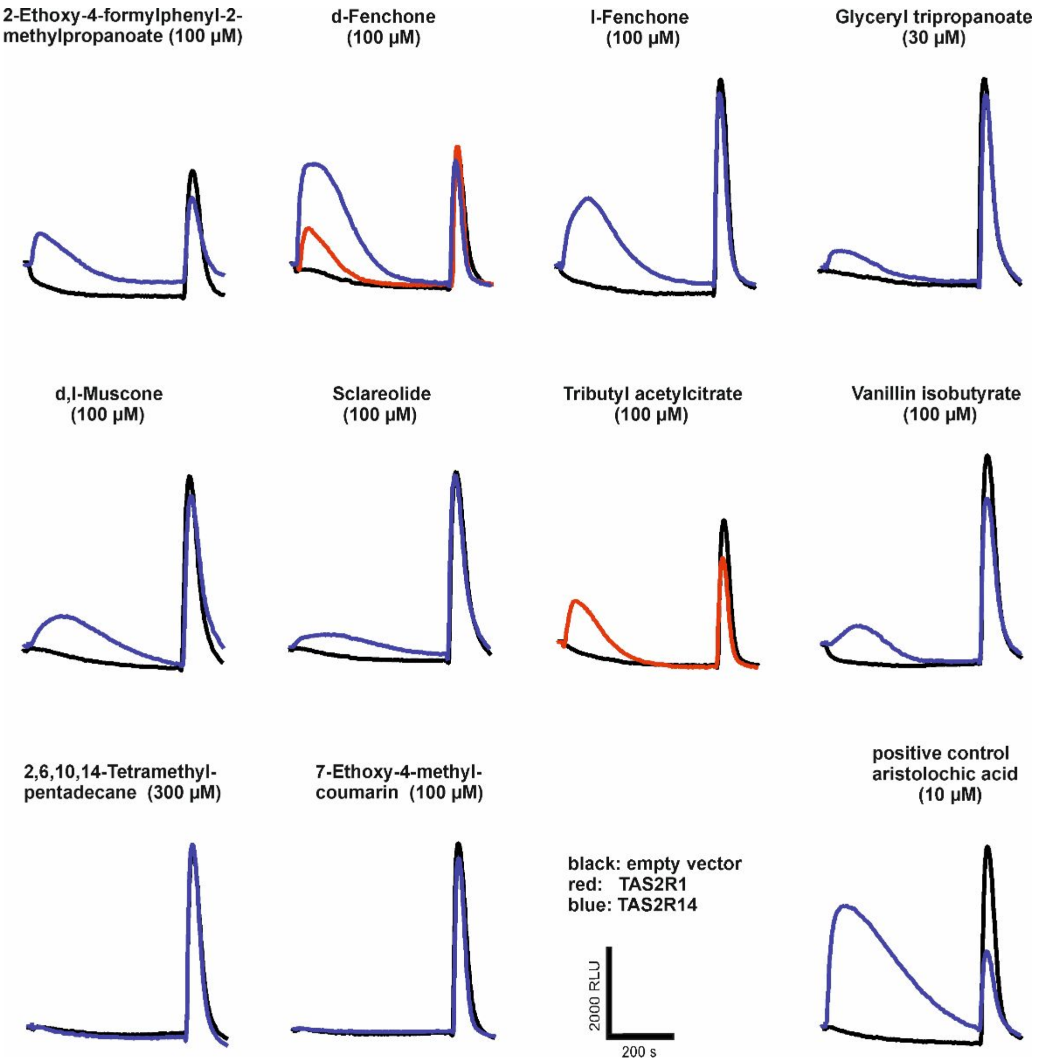


**Figure S1.** ***Screening of TAS2Rs with odorants.*** *Representative raw fluorescence traces of TAS2R14 (blue), TAS2R1 (red) or mock transfected cellular responses are shown. TAS2R14 transfected cells stimulated with 10 µM aristolochic acid served as positive control (bottom right trace). The second peak in each trace shows the cellular responses of endogenous somatostatin receptors stimulated with 100 nM somatostatin-14 serving as viability control. Scale bar: y-axis, relative light units (RLU); x-axis, time (seconds).*

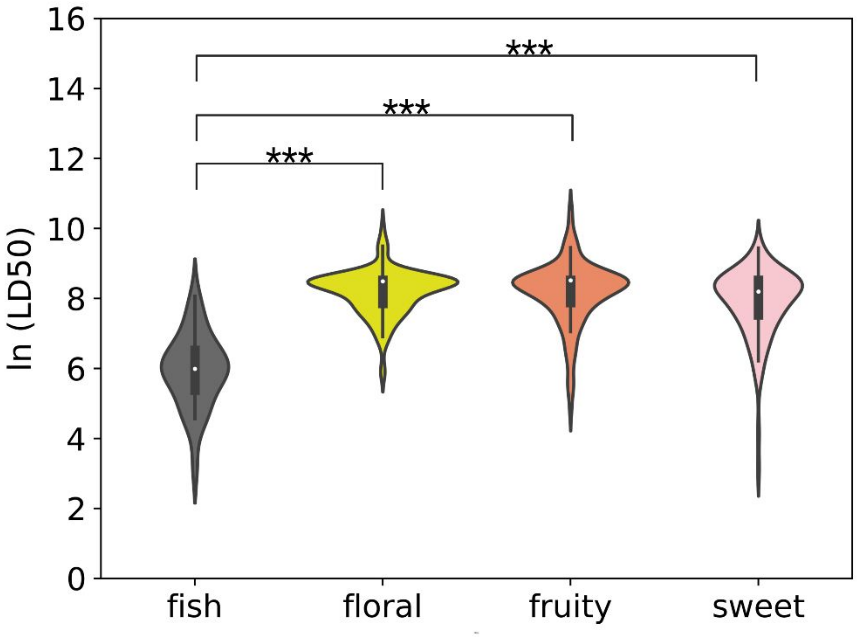


**Figure S2. *Comparison between ln($LD_{50}$ ) values of fishy smelling odorants (grey) and floral (yellow), fruity (orange), and sweet (pink) odorants*.** *A significant difference was observed using Dunnett's test, $P < 0.05$. *** $P < 0.0001$.*

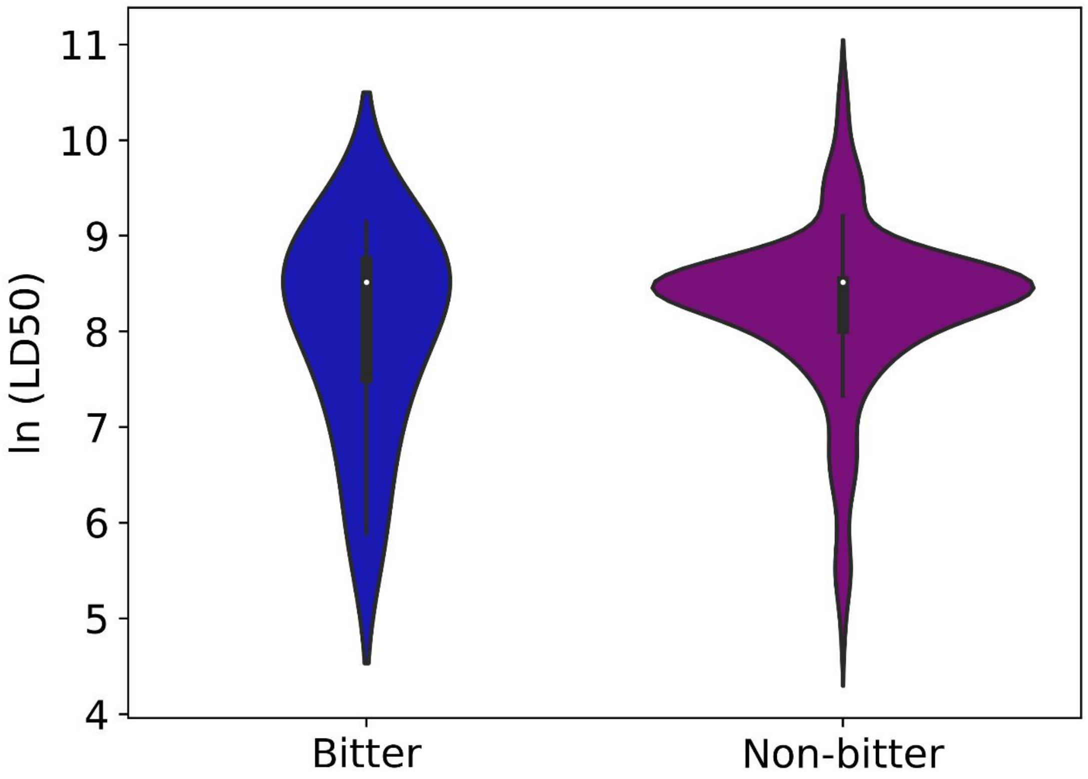


**Figure S3.** ***Comparison between ln(LD50) values of pleasant-smelling odorants with a bitter predicted taste (blue), non-bitter predicted taste (purple).*** *No significant difference was observed using a two-tailed t-test, P < 0.05.*

**Table S1.** Test compounds, their purity and suppliers.

| No. | compound | CAS | purity & suppliers |
|---|---|---|---|
| 1 | 2-Ethoxy-4-formyl phenyl 2-methylpropanoate | 188417-26-7 | 98%, BLD Pharmatech Ltd. |
| 2 | 7-Ethoxy-4-methylcoumarin | 87-05-8 | 98%, AP OLLD Scientific |
| 3 | d-Fenchone | 4695-62-9 | ≥98%, Sigma-Aldrich |
| 4 | l-Fenchone | 7787-20-4 | ≥98%, Sigma-Aldrich |
| 5 | Glyceryl tripropanoate | 139-45-7 | 97%, Thermo Scientific |
| 6 | d,l-Muscone | 541-91-3 | ≥95%, MP Biomedicals |
| 7 | Sclareolide | 564-20-5 | 97%, Sigma-Aldrich |
| 8 | 2,6,10,14- Tetramethylpentadecane | 1921-70-6 | 95%, Ehrenstorfer GmbH |
| 9 | Tributyl acetylcitrate | 77-90-7 | 98%, Sigma-Aldrich |
| 10 | Vanillin isobutyrate | 20665-85-4 | ≥98%, Sigma-Aldrich |

**Table S2.** Bitter Predicted odorants (high confidence)

| | |
|---|---|
| Acetin | CC(=O)OCC(COC(=O)C)OC(=O)C |
| 2-Acetyl-3,5-dimethylpyrazine | CC(=O)c1ncc(C)nc1C |
| 2-Acetyl-1,4,5,6-tetrahydropyridine | CC(=O)C1=CCCCN1 |
| 2-Acetyl-3,4,5,6-tetrahydropyridine | CC(=O)C1=NCCCC1 |
| Acetylthiazolidine | CC(=O)C1NCCS1 |
| Ammonia | N |
| Ammonium hydrogen sulphide | [NH4+].S |
| Ammonium hydroxide | [NH4+].[OH-] |
| Ammonium sulfide | [NH4+].[S-2] |
| Benzyl isothiocyanate | S=C=NCc1ccccc1 |
| Bismethyltetrahydrofurylspirotrithiolane | CC1OCCC12SSC3(CCOC3C)S2 |
| sec-Butylamine | CC[CH](C)N |
| Butylmethylpyrrolidinodithiazine | CCCCC1SC(C)N2CC=CC2S1 |
| Calone | Cc1ccc2OCC(=O)COc2c1 |
| Carvone acetate | CC(=O)OCC(=C)C1CC=C(C)C(=O)C1 |
| Cyclohexyl anthranilate | Nc1ccccc1C(=O)OC2CCCCC2 |
| Cyclohexyl formate | O=COC1CCCCC1 |
| 2,5-Dimethyl-4-(1-pyrrolidinyl)-3(2H)furanone | CC1OC(=C(N2CCCC2)C1=O)C |

| | |
|---|---|
| Dehydromenthofurolactone | C[CH]1CCC2=C(C)C(=O)OC2=C1 |
| (S)-8,9-dehydrotheaspirone | CC1=CC[C]2(O1)C(=CC(=O)CC2(C)C)C |
| Diethylamine | CCNCC |
| Dihydroactinidiolide | CC1(C)CCC[C]2(C)OC(=O)C=C12 |
| Dihydrotriethyldithiazine | CCC1NC(CC)SC(CC)S1 |
| Dihydrotrismethylpropyldithiazine | CC(C)CC1NC(CC(C)C)SC(CC(C)C)S1 |
| Dimethyl menthyl succinamide | CC(C)[C@@H]1CC[C@@H](C)C[C@H]1OC(=O)CCC(=O)N(C)C |
| Dimethylamine | CNC |
| Dimethylphenethylamine | C[CH](N(C)C)c1ccccc1 |
| Dimethylpyrrolidinodithiazine | C[CH]1S[CH](C)N2CCC[CH]2S1 |
| Ethoxymethylcoumarin | CCOc1ccc2C(=CC(=O)Oc2c1)C |
| Ethyl acetate | CCOC(=O)C |
| Ethyl formate | CCOC=O |
| Ethyl maltol isobutyrate | O=C(OC=1C(=O)C=COC=1CC)C(C)(C)[H] |
| 2-Ethoxy-4-formyl phenyl 2-methylpropanoate | CCOc1cc(C=O)ccc1OC(=O)C(C)C |
| Ethylamine | CCN |
| d-Fenchone | CC12CC[C@H](C1)C(C)(C)C2=O |
| l-Fenchone | C[C@@]12CC[C@@H](C1)C(C)(C)C2=O |
| Geranyl anthranilate | CC(=CCC\C(=C\COC(=O)c1ccccc1N)\C)C |
| Glyceryl tripropanoate | CCC(=O)OCC(COC(=O)CC)OC(=O)CC |
| Heliotropin propylene glycol acetal | CC1COC(O1)c2ccc3OCOc3c2 |
| Hexalactam | O=C1CCCCCN1 |
| Hexylamine | CCCCCCN |
| Hydroxypyridine | Oc1ccccn1 |
| Indole | c1ccc2[nH]ccc2c1 |
| Isobutyl anthranilate | CC(C)COC(=O)c1ccccc1N |
| Isobutylamine | CC(C)CN |
| Isobutyldimethyldihydrodithiazine | CC(C)CC1S[CH](C)N[CH](C)S1 |
| Isodecyl acetate | CC(C)CCCCCCCOC(=O)C |
| Isopentylamine | CC(C)CCN |
| Isopropanolamine | C[CH](O)CN |
| Isopropylamine | CC(C)N |
| Isoquinoline | c1ccc2cnccc2c1 |
| Methyl acetate | COC(=O)C |
| Methyl formyloxydodecanoate | CCCCCCCC(CCCC(=O)OC)OC=O |
| Methylamine | CN |
| Methylbutylamine | CC[CH](C)CN |
| 1-Methylpiperidine | CN1CCCCC1 |

| | |
|---|---|
| 2-Methylpiperidine | C[CH]1CCCCN1 |
| Methylpyrrolidine | CN1CCCC1 |
| Methylthiazolidine | C[CH]1NCCS1 |
| Methylthiopropylamine | CSCCCN |
| d,l-Muscone | CC1CCCCCCCCCCCCC(=O)C1 |
| Oxotetradecanoic acid glyceride | CCCCCCCCCCCC(=O)CC(=O)OC[CH](O)CO |
| Pentylamine | CCCCCN |
| 1-Amino-2-phenylethane | NCCc1ccccc1 |
| Piperazine | C1CNCCN1 |
| Piperidine | C1CCNCC1 |
| Propionylpyrroline | CCC(=O)C1=CCCN1 |
| Propiosyringone | CCC(=O)c1cc(OC)c(O)c(OC)c1 |
| Propyl acetate | CCCOC(=O)C |
| Propylamine | CCCN |
| Propylthiazolidine | CCC[CH]1NCCS1 |
| Pyrazine | c1cnccn1 |
| Pyridine | c1ccncc1 |
| Pyrrole | c1cc[nH]c1 |
| Pyrrolidine | C1CCNC1 |
| Pyrroline | C1CC=NC1 |
| Sclareolide | CC1(C)CCC[C@@]2(C)[C@H]1CC[C@@]3(C)OC(=O)C[C@H]23 |
| Terpinyl anthranilate | CC1=CC[CH](CC1)C(C)(C)OC(=O)c2ccccc2N |
| Tetrahydrotrimethylthiadiazine | CC1NC(C)SC(C)N1 |
| Tributyl acetylcitrate | CCCCOC(=O)CC(CC(=O)OCCCC)(OC(=O)C)C(=O)OCCCC |
| Trimethyldihydrodithiazine | C[CH]1N[CH](C)SC(C)S1 |
| Tyramine | NCCc1ccc(O)cc1 |
| Vanillin isobutyrate | COc1cc(C=O)ccc1OC(=O)C(C)C |
| Vanillin menthoxypropanediol acetal | COc1cc(ccc1O)[C@@H]2OC[C@@H](CO[C@H]3C[C@H](CC[C@@H]3C)C(C)C)O2 |
| Vomifoliol | C[C@@H](O)\C=C\[C@@]1(O)C(=CC(=O)CC1(C)C)C |
| WS-5 | CCOC(=O)CNC(=O)[C@@H]1C[C@H](C)CC[C@H]1C(C)C |

**Table S3.** Very Bitter Predicted odorants

| Name | SMILES |
|---|---|
| Carvone acetate | CC(=O)OCC(=C)C1CC=C(C)C(=O)C1 |
| Citral glyceryl acetal(s) | CC(=CCCC(=CC1OCC(O)CO1)C)C |
| Ethyl ricinoleate | CCCCCCC(O)C\C=C/CCCCCCCC(=O)OCC |
| Glyceryl monooleate | CCCCCCCC\C=C/CCCCCCCC(=O)OC[C@H](O)CO |
| Lactic acid | C[CH](O)C(=O)O |
| Methyl jasmonate | CC\C=C/C[CH]1[CH](CC(=O)OC)CCC1=O |

| | |
|---|---|
| Oxohexadecanoic acid glyceride | CCCCCCCCCCCCCC(=O)CC(=O)OC[CH](O)CO |
| Oxotetradecanoic acid glyceride | CCCCCCCCCCCC(=O)CC(=O)OC[CH](O)CO |
| Sorbitan monostearate | CCCCCCCCCCCCCCCCCC(=O)OCC(O)[C@H]1OC[C@@H](O)[C@@H]1O |
| Vanillin menthoxypropanediol acetal | COc1cc(ccc1O)[C@@H]2OC[C@@H](CO[C@H]3C[C@H](CC[C@@H]3C)C(C)C)O2 |

**Table S4.** Odorous-predicted bitterants list (from BitterDB):

| BitterDB ID | canonical_smiles | name |
|---|---|---|
| 15 | C1=CC=C(C=C1)C=O | benzaldehyde |
| 37 | C1=CC=C(C=C1)C(C(=O)C2=CC=CC=C2)O | Benzoin |
| 49 | C1=CC=C2C(=C1)C=CC(=O)O2 | Coumarin |
| 51 | CCCCCCCC=CC(C#CC#CC(C=C)O)O | Falcarindiol |
| 57 | C1=CC=C(C=C1)C(=O)N | Benzamide |
| 61 | CN(C)CCOC(C1=CC=CC=C1)C2=CC=CC=C2 | Diphenhydramine |
| 64 | C=CS(=O)C=C | Divinyl sulfoxide |
| 77 | CN1CCC23CCCCC2C1CC4=C3C=C(C=C4)OC | dextromethorphan |
| 90 | CC1C2CC2(CC1=O)C(C)C | (-)-alpha Thujone |
| 108 | C=CCN=C=S | allyl isothiocyanate |
| 109 | C1CCC(=O)NCC1 | Caprolactam |
| 110 | CN(C)C=S | dimethylthioformamide |
| 111 | CCC1=NC=CN=C1 | Ethylpyrazine |
| 118 | C1=CC=C(C=C1)CCN=C=S | phenethyl isothiocyanate |
| 130 | CC1=CC=CC=C1C(C2=CC=CC=C2)OCCN(C)C | orphenadrine |
| 134 | CC(C(C1=CC=CC=C1)O)N(C)CC=CC2=CC=CC=C2 | Cinnamedrine |
| 161 | CC(CNC)C1CCCCC1.Cl | Cyclexedrine Hydrochloride |
| 165 | CC(CC1CCCC1)NC.Cl | Cyclopentamine Hydrochloride |
| 188 | CS(=O)C | Dimethyl Sulfoxide |
| 192 | C1=CN=CC=C1C2=CC=NC=C2 | 4,4'-Bipyridine |
| 214 | CC1=CC=C(C=C1)F | Fluorotoluene |
| 217 | C1=COC(=C1)CO | Furfuryl Alcohol |
| 228 | C(CC#N)CC#N | Glutaronitrile |
| 232 | CC1=NC=CC2=C1NC3=CC=CC=C23 | Harman |
| 248 | OO | Hydrogen Peroxide |
| 275 | CC(C)O | Isopropyl Alcohol |
| 290 | CC1CC23CCCN4C2(C1)C(CCC4)C(=O)CC3 | Lycopodine |
| 299 | CC1CCC(C(=O)C1)C(C)C | Menthone |
| 313 | CC(CC1=CC=CC=C1)NC.Cl | Methamphetamine Hydrochloride |
| 369 | C(CCO)CCO | 1,5-Pentanediol |

| | | |
|---|---|---|
| 440 | C1=CNN=C1 | Pyrazole |
| 443 | CCC12CCCN(C1)CCC3=C(CC2)NC4=CC=CC=C3 4 | Quebrachamine |
| 449 | C1=CC=C2C(=C1)C=NC=N2 | Quinazoline |
| 511 | CC[N+](CC)(CC)CC.[OH-] | Tetraethylammonium Hydroxide |
| 546 | CCCC(CCC)C(=O)N | Valpromide |
| 588 | CC1=CC(=O)CC(C1)C(C)C | Homocamfin |
| 650 | CN1CCCC1C2=CN=CC=C2 | nicotine |
| 667 | C1CCNCC1 | piperidine |
| 673 | CCC=CCC=CCC=CCCCCCCCC(=O)O | alpha-Linolenic Acid |
| 684 | CCOC(=O)C1=CC=CC=C1 | Ethyl Benzoate |
| 685 | CC1=CC=CC=N1 | Picoline |
| 686 | CC(C)CNCC(C)C | Diisobutylamine |
| 687 | C1CCCC(=O)CCC1 | Cyclooctanone |
| 693 | C1CCN2CCCC(C2C1)CO | Lupinine |
| 694 | CN(C)CCC=C1C2=CC=CC=C2COC3=CC=CC=C3 1 | Doxepin |
| 695 | CC(=S)NC1=CC=CC=C1 | Thioacetanilide |
| 701 | CC1CCCC(N1)C | 2,6-Dimethylpiperidine |
| 702 | CN1C(=O)C=CC2=CC=CC=C21 | 1-methy-2-quinolinone |
| 711 | COC1=C(C=C(C=C1)CC=C)OC | Eugenyl methyl ether |
| 713 | CC(=O)C1=CC=CC=C1 | Acetophenone |
| 714 | CC(=O)C1=CC=C(C=C1)OC | Acetanisole |
| 715 | CC(C)(CC1=CC=CC=C1)O | A,a-dimethylphenethyl alcohol |
| 716 | CCOC(=O)CC1=CC=CC=C1 | Ethyl phenylacetate |
| 717 | CCC(=O)OCCC(C)C | Isoamyl propionate |
| 718 | CC(C)CCCC(C)CCO | 3,7-dimethyl-1-octanol |
| 719 | CC(CCO)O | 1,3-butylene glycol |
| 720 | CCOC=O | Ethyl formate |
| 721 | CC(C)COC(=O)C | Isobutyl acetate |
| 723 | CC(=O)OCC1=CC=CC=C1 | Benzyl acetate |
| 724 | CCC(=O)OCCC(C)CCC=C(C)C | Citronellyl propionate |
| 725 | CC(CCC=C(C)C)CCO | D-citronellol |
| 726 | CCCCCCOC(=O)C | Hexyl acetate |
| 727 | COC1=CC=C(C=C1)OC | P-dimethoxybenzene |
| 728 | CCC(=O)OCC(C)C | Isobutyl propionate |
| 729 | CCCCCC(C)O | 2-heptanol |
| 730 | CCCCC(CC)O | 3-heptanol |
| 731 | CCC(=O)OC(C)C | Isopropyl propionate |
| 732 | CC1(C2CCC(C2)(C1=O)C)C | D-fenchone |
| 733 | CC1(C2CCC(C2)(C1O)C)C | Fenchyl alcohol |
| 736 | CC(C)CCOC(=O)C | Isoamyl acetate |

| | | |
|---|---|---|
| 738 | CC1(C2CCC1(C(=O)C2)C)C | D-camphor |
| 739 | CC=CC1=CC(=C(C=C1)OC)OC | Isoeugenyl methyl ether |
| 740 | CC(=CCCC(=CC=O)C)C | Citral (neral) |
| 741 | CC(=CCCC(=CCOC(=O)C)C)C | Geranyl acetate |
| 742 | CC(=CCCC(=CCOC=O)C)C | Geranyl formate |
| 743 | C1=CC=C(C=C1)C=CCO | Cinnamyl alcohol |
| 744 | C1=CC=C(C=C1)C=CCOC=O | Cinnamyl formate |
| 745 | CCC(=O)OCC=C(C)CCC=C(C)C | Geranyl propionate |
| 746 | C1=CC=C(C=C1)CC=O | Phenylacetaldehyde |
| 750 | C1=CC=C(C=C1)CCO | Phenethyl alcohol |
| 753 | CC(=O)OC | Methyl acetate |
| 754 | CC1=CC2=C(C=C1)OC(=O)C=C2 | 6-methylcoumarin |
| 755 | CC1=CC(=C(C=C1)O)OC | 2-methoxy-4-methylphenol |
| 756 | CC1=CCC(=CC1)C(C)C | P-mentha-1,4-diene |
| 757 | CC1=CC=C(CC1)C(C)C | 1-isopropyl-4-methyl |
| 758 | CC(C)C(=O)OCCC1=CC=CC=C1 | Phenethyl isobutyrate |
| 759 | CC(C)C(=O)OCCCC1=CC=CC=C1 | 3-phenylpropyl isobutyrate |
| 760 | C1=CC=C(C=C1)CCOC=O | Phenethyl formate |
| 761 | CCCOC(=O)CC | Propyl propionate |
| 762 | CCCOC(=O)C | Propyl acetate |
| 763 | CCCOC=O | Propyl formate |
| 764 | CCCCCCC(=O)C | 2-octanone |
| 767 | COC1=CC=CC=C1C=O | O-methoxybenzaldehyde |
| 768 | CC(C)CC(=O)OCCC1=CC=CC=C1 | Phenethyl isovalerate |
| 769 | CC(CCCC(=C)C)CCOC(=O)C | Rhodinyl acetate |
| 770 | CCCCCCCCCO | Nonyl alcohol |
| 771 | CCCCCCCCCOC(=O)C | Nonyl acetate |
| 773 | CC(=CCCC(=O)C)C | 6-methyl-5-hepten-2-one |
| 775 | CCCOC(=O)CC(C)C | Propyl isovalerate |
| 776 | CCC(C=C)O | 1-penten-3-ol |
| 777 | CC1=CCC(CC1)C(C)(C)OC=O | Terpinyl formate |
| 778 | CCC(C)C(=O)OC1CC(CCC1C(C)C)C | Menthyl isovalerate |
| 779 | CC(=O)OCCCC1=CC=CC=C1 | 3-phenylpropyl acetate |
| 780 | COC1=CC=C(C=C1)C=O | P-methoxybenzaldehyde |
| 781 | COC(CC1=CC=CC=C1)OC | Phenylacetaldehyde dimethyl acetal |
| 782 | CC(=CCCC(C)(C=C)OC=O)C | Linalyl formate |
| 783 | CC(CCCC(=C)C)CCOC=O | Rhodinyl formate |
| 786 | CCC(=O)OC(C)(C)C1CCC(=CC1)C | Terpinyl propionate |
| 787 | CC(C1=CC=CC=C1)OC(=O)C | A-methylbenzyl acetate |
| 790 | CC1=CCC(CC1)C(C)(C)OC(=O)CC(C)C | Terpinyl isovalerate |
| 791 | CC(=CCCC(=CCO)C)C | Nerol |

| | | |
|---|---|---|
| 793 | CCCCCCCCCCC=CC=O | 2-tridecenal |
| 794 | CC(C)CC(=O)OCC=C(C)CCC=C(C)C | Neryl isovalerate |
| 796 | CC(C)C(C=O)C1=CC=CC=C1 | 3-methyl-2-phenylbutyraldehyde |
| 797 | CC(C)CC(=O)OCCC(C)CCCC(=C)C | Rhodinyl isovalerate |
| 805 | C1CC2=CC=CC=C2C(=O)C1 | Alpha-tetralone |
| 839 | CC(C)C1=CC=C(C=C1)C=NO | 4-Isopropylbenzaldoxime |
| 868 | C1=CC=C(C=C1)C2=CC(=O)C3=CC=CC=C3O2 | flavone |
| 893 | C1C(OC2=CC=CC=C2C1=O)C3=CC=CC=C3 | flavanone |
| 902 | C1=CC=C(C=C1)C=CC(=O)C2=CC=CC=C2 | chalcone |
| 905 | C1=CC=C(C=C1)C=CC(=O)C2=CC=C(C=C2)O | 4-hydroxychalcone |
| 919 | C1=CC=C(C=C1)C2=COC3=CC=CC=C3C2=O | isoflavone |
| 925 | C1=CC=C2C(=C1)C(=O)C3=CC=CC=C3O2 | xanthonee |
| 926 | COC1=CC=C(C=C1)O | 4-hydroxyanisol |
| 929 | C1=CC=C2C(=C1)C=CC=C2C(=O)O | 1-Naphthoic acid |
| 946 | CC1=CCCC(=CC2C(CC1)C(=C)C(=O)O2)C | costunolide |
| 955 | C1CCN2CC3CC(C2C1)CN4C3CCCC4 | sparteine |
| 961 | CC12CCC(CC1CCC3C2CCC4(C3CCC4=O)C)O | Androsterone |
| 964 | CC(C)CCCCCCCC=CC(=O)O | cis-11-Methyl-2-dodecenoic acid |
| 972 | C1=CC=C(C(=C1)O)O | pyrocatechin |
| 974 | C1=CC=C(C=C1)CCN=C=O | phenylethyl isothiocyanate |
| 1006 | CCCCCCCC=CCC#CC#CC(C=C)O | falcarinol |
| 1112 | CC1CC2=C(C1=O)NCCC=C2 | 7-methyl-2,3,6,7-tetrahydrocyclopenta[b]azepin-8(1H)-one |
| 1176 | CC1CCC(C(C1)O)C(C)C | L-menthol |
| 1223 | C1CC2C=CC1C=C2C=NO | (E)-1-(Bicyclo[2.2.2]octa-2,5-dien-2-yl)-N-hydroxymethanimine |
| 1228 | C1=CC=C2C(=C1)C(=CN2)CCO | 2-(1H-indol-3-yl)ethanol |
| 1298 | C1=CN=CN1 | imidazole |
| 1299 | C1=CC=NN=C1 | Pyridazine |
